# Are We Ready for Nosocomial *Candida auris* Infections? Rapid Identification and Antifungal Resistance Detection Using MALDI-TOF Mass Spectrometry May Be the Answer

**DOI:** 10.3389/fcimb.2021.645049

**Published:** 2021-03-16

**Authors:** Elena De Carolis, Federica Marchionni, Marilisa La Rosa, Jacques F. Meis, Anuradha Chowdhary, Brunella Posteraro, Maurizio Sanguinetti

**Affiliations:** ^1^Dipartimento di Scienze di Laboratorio e Infettivologiche, Fondazione Policlinico Universitario A. Gemelli IRCCS, Rome, Italy; ^2^Department of Medical Microbiology and Infectious Diseases, Canisius Wilhelmina Hospital, Nijmegen, Netherlands; ^3^Centre of Expertise in Mycology, Radboudumc/Canisius Wilhelmina Hospital, Nijmegen, Netherlands; ^4^Vallabhbhai Patel Chest Institute, Department of Medical Mycology, University of Delhi, Delhi, India; ^5^Dipartimento di Scienze Biotecnologiche di Base, Cliniche Intensivologiche e Perioperatorie, Università Cattolica del Sacro Cuore, Rome, Italy

**Keywords:** *Candida auris*, 3-hour MS-AFST, multidrug resistance, anidulafungin, new emerging pathogen, rapid identification and susceptibility testing

## Abstract

The occurrence of multidrug-resistant *Candida auris* isolates and the increased mortality associated with invasive infections or outbreaks due to this *Candida* species have been reported in many healthcare settings. Therefore, accurate and rapid identification at the species level of clinical *C. auris* isolates as well as their timely differentiation as susceptible or resistant to antifungal drugs is mandatory. Aims of the present study were to implement the MALDI-TOF mass spectrometry (MS) Bruker Daltonics Biotyper^®^ database with *C. auris* spectrum profiles and to develop a fast and reproducible MS assay for detecting anidulafungin (AFG) resistance in *C. auris* isolates. After creation of main *C. auris* spectra, a score-oriented dendrogram was generated from hierarchical cluster analysis, including spectra of isolates from *C. auris* and other *Candida* (*C. glabrata*, *C. guilliermondii*, *C. haemulonii*, *C. lusitaniae*, and *C. parapsilosis*) or non-*Candida* (*Rhodotorula glutinis*) species. Cluster analysis allowed to group and classify the isolates according to their species designation. Then, a three-hour incubation antifungal susceptibility testing (AFST) assay was developed. Spectra obtained at null, intermediate, or maximum AFG concentrations were used to create composite correlation index matrices for eighteen *C. auris* isolates included in the study. All six resistant *C. auris* isolates were detected as resistant whereas 11 of 12 susceptible *C. auris* isolates were detected as susceptible by the MS-AFST assay. In conclusion, our MS-based assay offers the possibility of rapidly diagnosing and appropriately treating patients with *C. auris* infection.

## Introduction

The fungal pathogen *Candida auris* has emerged over a decade ago in East Asia and, since then, multidrug-resistant *C. auris* isolates causing nosocomial outbreaks have been isolated in many countries worldwide (Du et al., 2020). This is alarming because bloodstream infections caused by *C. auris* have been associated with a 30 to 60% rate of infection-related mortality (Chowdhary et al., 2017). To cope with the healthcare issues arisen from this emerging pathogen, species-level identification of *C. auris* isolates and their differentiation as susceptible or resistant to the commonly used antifungals agents became mandatory. However, biochemical/enzymatic identification methods are time-consuming and, in the beginning, misidentified *C. auris* (Kathuria et al., 2015); whereas MALDI-TOF mass spectrometry (MS) based identification was not optimal (Buil et al., 2019) until when MALDI-TOF MS databases were enriched with *C. auris* specific mass spectrum profiles (Girard et al., 2016; Bao et al., 2018). Of course, molecular methods such as *C. auris* colony-specific PCR and DNA sequencing (Kordalewska et al., 2017; Valentin et al., 2018) are efficient but less rapid than those based on the MALDI-TOF MS analysis.

While defined CLSI or EUCAST minimum inhibitory concentration (MIC) breakpoints for *C. auris* susceptibility are unavailable to date (Kordalewska and Perlin, 2019), tentative MIC breakpoints have been proposed (Lockhart et al., 2017). Nonetheless, 99% of *C. auris* isolates studied by Arendrup et al. had high MIC values to fluconazole while occasionally retaining full susceptibility to other triazole antifungal agents (Arendrup et al., 2017). Regarding echinocandins or amphotericin B, resistance rates are variable, being 7% and 10 to 35%, respectively, whereas acquired resistance to echinocandins has been associated with the presence of S639F orS639P mutations in the glucan-synthase encoding gene *FKS1*, which is the target of echinocandins (Kordalewska et al., 2018). While echinocandins are regarded as first-line treatment for *C. auris* infections, echinocandin-resistant isolates of *C. auris* may occur in patients during antifungal treatment (Park et al., 2019; Novak et al., 2020), calling for repeated susceptibility testing in order to monitor possible therapeutic failures.

Consistent with these observations, we implemented the MALDI Biotyper^®^ database (Bruker Daltonics, Bremen, Germany) with mass spectrum profiles from *C. auris* isolates, and we developed a fast and reproducible MALDI-TOF MS based assay to detect resistance to the echinocandin anidulafungin in *C. auris* isolates.

## Materials and Methods

### Study Isolates

The *C. auris* isolates used in this study were clinical clade I isolates that are part of a collection at the Centre of Expertise in Mycology, Nijmegen, The Netherlands. Additionally, a single *C. auris* isolate that was derived from the first diagnosed case of candidemia in Central Italy was also studied. In total, eight isolates that were confirmed to be *C. auris* by PCR and sequencing of the ribosomal DNA internal transcribed spacer (ITS) region were submitted to protein extraction according to a previously developed MALDI-TOF MS protocol (De Carolis et al., 2014). Briefly, yeast cells were suspended in 10% formic acid and then vortexed; one µL of lysate was placed on the MALDI target plate to obtain 12 technical replicates, which were overlaid each with one µL of absolute ethanol before allowing co-crystallization with the α-cyano-4-hydroxycinnamic acid matrix (Bruker Daltonics). A total of ≥5000 laser shots were used to generate a main spectrum profile (MSP) for each isolate, which was then added to the Bruker MALDI Biotyper^®^ database. Isolates were also submitted to antifungal susceptibility testing (AFST), which was performed using a MALDI-TOF MS based assay (see below).

### MALDI-TOF MS Identification Analysis

The MALDI-TOF MS analysis on *C. auris* isolates was undertaken with a Microflex LT mass spectrometer, by which spectra were recorded in the positive linear mode within a 2000–20000 Da range. A bacterial test standard (BTS255343; Bruker Daltonics) was used for the instrument calibration. Preliminarily, the Bruker Biotyper^®^ database (version 7.0; 7311 entries), which contains only three MSP profiles of *C. auris*, did not allow reliable identification (i.e., log(score) values were lower than 2.0, which is the manufacturer-recommended cutoff level for MALDI-TOF MS species-level identification) of the isolates included in the study. Then, MSP profiles from the isolates were obtained and added to the Bruker Biotyper^®^ database—this resulted into an extended MALDI database—following Bruker Daltonics standard operating procedures (https://spectra.folkhalsomyndigheten.se). Accordingly, isolates’ protein extracts were prepared submitting each isolate—which grew on Sabouraud dextrose agar for 48 h at 37°C—to the aforementioned fast formic-acid extraction procedure. High-quality mass spectra from different spots for each isolate were analyzed by the MALDI BioTyper^®^ software, and used to create a MSP for each isolate. After MSP creation, using the integrated statistical tool Matlab 7.1 (The MathWorks Inc.; Natick, MA, USA), a hierarchical cluster analysis was performed to generate a score-oriented dendrogram, which included MSP profiles from *C. auris* isolates together with those from isolates of other *Candida* (*C. glabrata*, *C. guilliermondii*, *C. haemulonii*, *C. lusitaniae*, and *C. parapsilosis*) or non-*Candida* (*Rhodotorula glutinis*) species.

Then, mass spectrum profiles from 18 challenge isolates were analyzed in duplicate, automatically acquired, and matched against those of the extended MALDI database to allow species-level identification, for which the highest log(score) value from any match was reported. Finally, the challenge isolates were matched against an updated Bruker Biotyper^®^ database (version 9.0; which includes nine *C. auris* isolates), as well as against the Bruker-CDC merged MicrobeNet database (version 9978; https://microbenet.cdc.gov/).

### MALDI-TOF MS Antifungal-Resistance Detection Analysis

According to our previous studies (De Carolis et al., 2012; Vella et al., 2013; Vella et al., 2017), selected *C. auris* isolates were exposed to serial AFG concentrations (i.e., ranging from 0.06 to 512 μg/mL) and to a null concentration (0 μg/mL) for 3 h at 37°C. The spectrum obtained at each concentration was matched against those at the two extreme concentrations, i.e., null (0 μg/mL) and maximum (512 μg/mL), respectively. Values resulting from the composite correlation index (CCI) matrices derived from the spectra indicated a clear diversity between the spectra when values were near 0 and high similarity between the spectra when values were around 1. These experiments allowed to find a breakpoint AFG concentration, which was used in a subsequent assay. For each isolate, two technical and three biological replicates were analyzed for both the preliminary experiments and the assays illustrated below. Briefly, *C. auris* cells (1 × 10^7^ CFU/mL, as determined by cell counting) were exposed to AFG concentrations of 64 μg/mL (maximum), 0.06 μg/mL (breakpoint), and 0 μg/mL (null) for 3 hours at 37°C under agitation (300 rpm) in RPMI-1640 medium (supplemented with L-glutamine and sodium bicarbonate; R8758; Merck, Rome, Italy). Cells were centrifuged and the pellet washed twice with deionized water before the resuspension in 10% formic acid. The *C. auris* isolates profiles obtained at null, intermediate, or maximum AFG concentrations were used to create CCI matrices within the range 3000–8000 Da (15 intervals) using the MALDI Biotyper 3.1 software (De Carolis et al., 2012).

As previously reported, we matched each “breakpoint” spectrum against the spectrum at the maximum concentration or the spectrum at the null concentration of AFG. Then, isolates were classified as susceptible or resistant to AFG when the CCI value obtained matching the breakpoint spectrum with the “maximum” spectrum was higher or lower than the spectrum obtained matching the breakpoint spectrum with the “null” spectrum, respectively. The CCI ratios were calculated dividing the CCI_max_ by the CCI_null_, and a *C. auris* isolate was categorized as susceptible if the CCI_max_/CCI_null_ ratio was >1 or as resistant if the CCI_max_/CCI_null_ ratio was <1. Results of the mass spectrometry AFST (MS-AFST) were compared with the MIC values obtained using the commercial AFST method Sensititre YeastOne (Thermo Scientific, Italy), which was an adaptation of the CLSI M27-A3 broth microdilution standard (CLSI, 2008). The tentative MIC breakpoints (expressed as µg/mL) above mentioned were used as criteria to interpret AFST results.

## Results and Discussion

**Figure 1:**
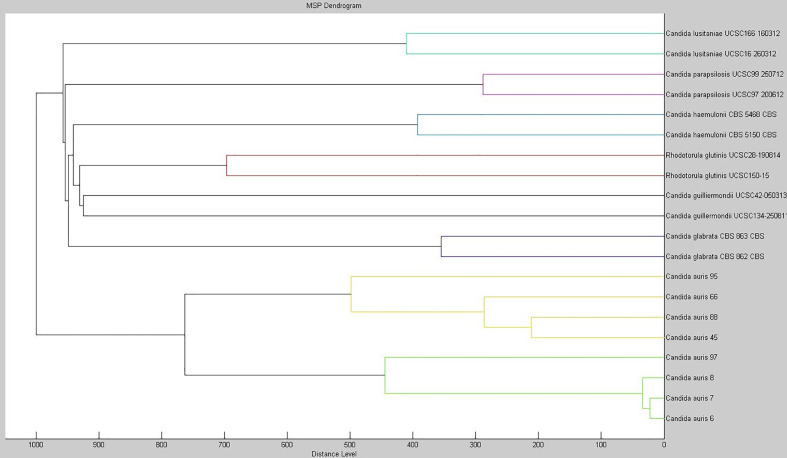
Dendrogram created with the MSPs from eight isolates of *C. auris* and from two isolates each of *C. glabrata*, *C. guilliermondii*, *C. haemulonii*, *C. lusitaniae*, *C. parapsilosis*, and *Rhodotorula glutinis*. The distance values are normalized to the maximum value of 1000.

As of November 2019, we identified in our hospital a bloodstream infection due to *C. auris*, which represented the first case of invasive *C. auris* infection detected in Central Italy where the hospital is located. *Ad hoc* extending the MALDI Bruker Biotyper^®^ database version 7.1 with MSPs from *C. auris* isolates enabled us to identify the bloodstream *Candida* pathogen as *C. auris*, which yielded a MALDI log(score) of 2.230. Using the extended database as well as the Bruker-CDC merged database (data not shown), we were able to obtain reliable species-level identification (log(score) values, >2.0) for 18 C*. auris* isolates from clade I. Particularly for *C. auris*, phenotypic identification methods such as VITEK 2 YST, API 20C, or BD Phoenix systems as well as the fully automated MicroScan system fail to provide accurate identification at the species level. As shown in this and in other studies (Delavy et al., 2019), older versions of commercially available MALDI-TOF MS databases proved to be unable to identify *C. auris*. To corroborate our findings, we investigated the relatedness among 20 MSPs obtained from eight isolates of *C. auris* and two isolates each of *C. glabrata*, *C. guilliermondii*, *C. haemulonii*, *C. lusitaniae*, *C. parapsilosis*, and *Rhodotorula glutinis*. **Figure 1** shows the score-oriented dendrogram resulting from the hierarchical cluster analysis used for this investigation. Based on correlation distance values, all the *C. auris* isolates grouped in a separate branch of the dendrogram according to their species designation.

**Figure 2:**
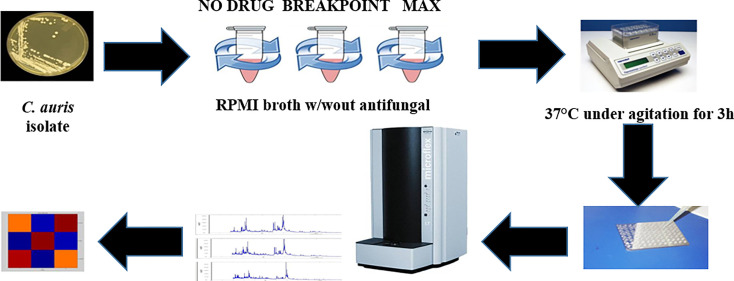
MS-AFST assay for resistance detection in *C. auris*. Shown is the workflow that includes an incubation with AFG at 37°C for 3 hours, followed by a generation of mass spectrum profiles that are visualized in a CCI matrix (heat map).

**Figure 3:**
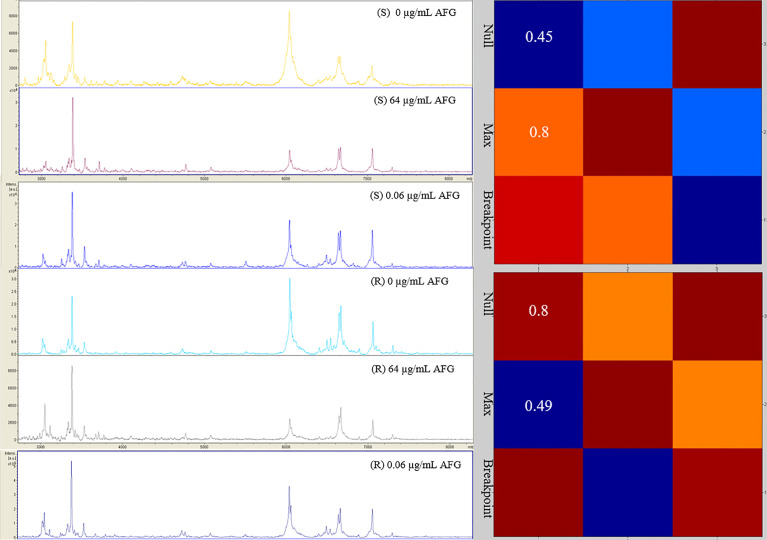
Raw mass spectra of susceptible (*n* = 1) and resistant (*n* = 1) *C. auris* isolates incubated at the null, maximum, or breakpoint concentration of AFG. In the heat map, hot colors denote similar spectra whereas cold colors denote dissimilar spectra. CCI values are reported accordingly.

In parallel, we investigated the capability of an MS-AFST assay to detect susceptibility or resistance phenotypes for 18 C*. auris* isolates exposed to 64 μg/mL, 0.06 μg/mL, or 0 μg/mL concentrations of AFG, respectively, for three hours at 37°C (**Figure 2**). Although MALDI-TOF MS-based AFST assays have already been developed for several antifungal drugs and *Candida* species, including *C. auris* (Delavy et al., 2019; Vatanshenassan et al., 2019), we defined the optimal AFG concentrations for use with *C. auris*. Details of our MS-AFST assay are shown in **Figure 3**, where the relationship between *C. auris* mass spectra acquired at the indicated AFG concentrations was visualized in a matrix (heat map) of CCI values and the relative MALDI-TOF MS profiles are reported for a susceptible and resistant *C. auris* isolate.

According to MIC interpretive criteria, *C. auris* isolates were identified as susceptible or resistant to AFG, respectively. As shown in **Table 1**, we found that the MS-AFST assay correctly classified 6 (100%) of 6 resistant isolates and 11 (91.7%) of 12 susceptible isolates, as determined by the Sensititre YeastOne (herein used as the reference method) and published tentative echinocandin breakpoints (Lockhart et al., 2017). Note that one *C. auris* isolate that had an intermediate AFG susceptibility (MIC value, 1 µg/mL) was classified as resistant by the AFST-MS assay. Although *C. auris* isolates displaying a wild-type *FKS1* hot spot 1 (HS1) genotype are relatively frequent (Chowdhary et al., 2018), we noted that two of six resistant isolates (with elevated AFG MICs) harbored the S639F mutation in the *FKS1* HS1 region. Very recently, Sharma et al. (2020) investigated a possible role for upregulated chitin or cell-wall stress response genes in echinocandin-resistant and -intermediate C. *auris* isolates. Despite being beyond the study’s scope, it would have been interesting to investigate whether the *C. auris* isolate showing intermediate susceptibility by the Sensititre YeastOne method but resistance with the MS-AFST assay could have any altered expression in specific genes related to AFG-susceptible or -resistant phenotypes. Conversely, we did not exclude the hypothesis that an additional single-nucleotide polymorphism (i.e., outside HS1 region) might have attenuated the otherwise resistant phenotype in that isolate.

**Table 1:** MS-AFST categorization of wild-type (WT) or non-WT *C. auris* isolates tested against anidulafungin.

Isolate designation	*FKS1* phenotype		Anidulafungin susceptibility testing					
			CLSI		MS-AFST	CCI ratio	CCI_null_	CCI_max_
			MIC (µg/mL)	Category				
10.05.12.66	S639F		≥4	R	R	0.97	1.00	0.97
						0.95	1.00	0.95
						0.30	1.00	0.30
10.05.12.62	S639F		≥4	R	R	0.74	0.92	0.68
						0.69	0.80	0.55
						0.50	1.00	0.50
10.05.12.57	WT		≥4	R	R	0.58	0.81	0.47
						0.66	0.71	0.47
						0.66	0.73	0.48
10.05.12.59	WT		0.125	S	S	1.83	0.48	0.88
						1.78	0.45	0.80
						2.60	0.35	0.91
10.05.18.95	WT		≥4	R	R	0.85	0.97	0.82
						0.91	0.99	0.90
						0.92	0.37	0.34
10.05.12.45	WT		≥4	R	R	0.23	0.97	0.22
						0.80	1.00	0.80
						0.61	0.80	0.49
10.05.12.88	WT		1	S	R	0.83	0.60	0.50
						0.86	0.80	0.69
						0.73	0.81	0.59
10.05.18.97	WT		≥4	R	R	0.89	0.96	0.85
						0.95	0.95	0.90
						0.93	0.98	0.91
10.03.10.64	WT		0.06	S	S	5.45	0.11	0.60
						5.50	0.14	0.77
						1.30	0.70	0.91
10.03.10.63	WT		0.125	S	S	2.50	0.30	0.75
						2.75	0.20	0.55
						2.21	0.38	0.84
10.03.11.62	WT		0.125	S	S	2.21	0.39	0.86
						3.67	0.15	0.55
						2.57	0.21	0.54
10.08.12.28	WT		0.25	S	S	1.09	0.87	0.95
						1.44	0.55	0.79
						1.09	0.57	0.62
10.08.12.29	WT		0.25	S	S	0.99	0.84	0.83
						1.52	0.50	0.76
						1.11	0.35	0.39
10.05.15.49	WT		0.125	S	S	1.15	0.75	0.86
						1.38	0.13	0.18
						1.07	0.71	0.77
10.08.16.39	WT		0.125	S	S	1.05	0.19	0.20
						1.69	0.49	0.83
						1.86	0.37	0.69
10.08.12.66	WT		0.125	S	S	5.66	0.09	0.51
						5.10	0.10	0.51
						1.13	0.69	0.78
10.11.02.11	WT		0.06	S	S	1.01	0.95	0.96
						1.09	0.69	0.44
						1.10	0.59	0.65
10.08.12.39	WT		0.125	S	S	1.03	0.79	0.81
						0.98	0.81	0.79
						1.07	0.57	0.61

R, resistant; S, susceptible.

Our study has some limitations. The data set is somewhat limited not only by the size—which is understandable considering that *C. auris* is not a ubiquitous pathogen—but also by the geographic restriction—which is understandable considering that *C. auris* isolates used by us represented clade I. Thus, it is not surprising that our isolates gave MALDI-TOF MS identification (log)scores of <2.0 when challenged with the Bruker Biotyper^®^ database containing three isolates (version 7.0) or nine isolates (version 9.0). Likewise, it is not surprising that extending the Bruker Biotyper^®^ database with the MSPs from eight *C. auris* isolates, which had the same geographic origin as those being challenged, resulted in higher (log)scores—three *C. auris* isolates in the database version 7.0 were from Korea or Japan. Additionally, we generated MSPs from *C. auris* isolates using the same formic-acid based extraction method as that used to prepare mass spectra from the isolates being identified. Taken together, our experimental situation was such that we did not consider to lower MALDI-TOF MS (log)scores below 2.0 (i.e., to ≥1.7) for species-level identification, which is instead the strategy applied in many laboratories that perform identifications for *Candida*, *Aspergillus*, or difficult-to-identify bacteria. Particularly for filamentous fungi including *Aspergillus*, this strategy was shown to significantly increase the rate of accurate species-level identifications while not increasing the number of misidentifications even when cryptic *Aspergillus* species were tested (Wilkendorf et al., 2020).

Our study adds support to the successfully applied CCI-based proteomic approaches for antifungal resistance detection in *Candida* species, which offer the advantage to considerably reduce the time to result (three hours in our study) compared with conventional AFST methods (Delavy et al., 2019; Roberto et al., 2020). In addition to being a cost-effective method (few euro-cents per run), our MS-AFST assay differs from the growth-based MALDI Biotyper antibiotic (antifungal) susceptibility test rapid assay (MBT-ASTRA) recently developed for rapid detection of AFG-resistant *C. glabrata* and *C. auris* isolates (Vatanshenassan et al., 2018; Vatanshenassan et al., 2019). In particular, our assay can be even successful with poorly growing isolates, thereby avoiding the need to set an isolate-dependent growth cutoff. However, further studies are necessary to ascertain the usefulness of MALDI-TOF MS based assays for the management of outbreaks sustained by multidrug-resistant *C. auris* isolates in the hospital setting.

## Data Availability Statement

The original contributions presented in the study are included in the article/supplementary material. Further inquiries can be directed to the corresponding author.

## Author Contributions

EC developed the theory, analyzed the data and wrote the manuscript with support from BP and MS. FM and MR carried out the experiment. JM and AC provided critical feedback. All authors contributed to the article and approved the submitted version.

## Conflict of Interest

The authors declare that the research was conducted in the absence of any commercial or financial relationships that could be construed as a potential conflict of interest.

## References

[B1] Arendrup M. C. Prakash A. Meletiadis J. Sharma C. Chowdhary A. (2017). Comparison of EUCAST and CLSI reference microdilution MICs of eight antifungal compounds for *Candida auris* and associated tentative epidemiological cutoff values. Antimicrob. Agents Chemother. 61, e00485–e004 7. 10.1128/AAC.00485-17 28416539 PMC5444165

[B2] Bao J. R. Master R. N. Azad K. N. Schwab D. A. Clark R. B. Jones R. S.. (2018). Rapid, accurate identification of *Candida auris* by using a novel matrix-assisted laser desorption ionization-time of flight mass spectrometry (MALDI-TOF MS) database (library). J. Clin. Microbiol. 56, e01700–e01717. 10.1128/JCM.01700-17 29367296 PMC5869814

[B3] Buil J. B. van der Lee H. A. L. Curfs-Breuker I. Verweij P. E. Meis J. F. (2019). External quality assessment evaluating the ability of Dutch clinical microbiological laboratories to identify *Candida auris*. J. Fungi 5, 94. 10.3390/jof5040094 PMC6958413 31591307

[B4] Chowdhary A. Sharma C. Meis J. F. (2017). *Candida auris*: a rapidly emerging cause of hospital-acquired multidrug-resistant fungal infections globally. PloS Pathog. 13, e1006290. 10.1371/journal.ppat.1006290 28542486 PMC5436850

[B5] Chowdhary A. Prakash A. Sharma C. Kordalewska M. Kumar A. Sarma S.. (2018). A multicentre study of antifungal susceptibility patterns among 350 *Candida auris* isolates(2009–17) in India: role of the *ERG11* and *FKS1* genes in azole and echinocandin resistance. J. Antimicrob. Chemother. 73, 891–899. 10.1093/jac/dkx480 29325167

[B6] CLSI (2008). M27-A3 reference method for broth dilution antifungal susceptibility testing of yeasts; approved standard. 3rd (Wayne PA: Clinical and Laboratory Standards Institute).

[B7] De Carolis E. Vella A. Florio A. R. Posteraro P. Perlin D. S. Sanguinetti M.. (2012). Use of matrix-assisted laser desorption ionization-time of flight mass spectrometry for caspofungin susceptibility testing of *Candida* and *Aspergillus* species. J. Clin. Microbiol. 50, 2479–2483. 10.1128/JCM.00224-12 22535984 PMC3405623

[B8] De Carolis E. Vella A. Vaccaro L. Torelli R. Posteraro P. Ricciardi W.. (2014). Development and validation of an in-house database for matrix-assisted laser desorption ionization-time of flight mass spectrometry-based yeast identification using a fast protein extraction procedure. J. Clin. Microbiol. 52, 1453–1458. 10.1128/JCM.03355-13 24554755 PMC3993681

[B9] pDelavy M. Dos Santos A. R. Heiman C. M. Coste A. T. (2019). Investigating antifungal susceptibility in *Candida* species with MALDI-TOF MS-based assays. Front. Cell. Infect. Microbiol. 9, 19. 10.3389/fcimb.2019.00019 30792970 PMC6375026

[B10] Du H. Bing J. Hu T. Ennis C. L. Nobile C. J. Huang G. (2020). *Candida auris*: epidemiology, biology, antifungal resistance, and virulence. PloS Pathog. 16, e1008921. 10.1371/journal.ppat.1008921 33091071 PMC7581363

[B11] Girard V. Mailler S. Chetry M. Vidal C. Durand G. van Belkum A.. (2016). Identification and typing of the emerging pathogen *Candida auris* by matrix-assisted laser desorption ionisation time of flight mass spectrometry. Mycoses 59, 535–538. 10.1111/myc.12519 27292939

[B12] Kathuria S. Singh P. K. Sharma C. Prakash A. Masih A. Kumar A.. (2015). Multidrug-resistant *Candida auris* misidentified as *Candida haemulonii*: characterization by matrix-assisted laser desorption ionization-time of flight mass spectrometry and DNA sequencing and its antifungal susceptibility profile variability by Vitek 2, CLSI broth microdilution, and Etest method. J. Clin. Microbiol. 53, 1823–1830. 10.1128/JCM.00367-15 25809970 PMC4432077

[B13] Kordalewska M. Zhao Y. Lockhart S. R. Chowdhary A. Berrio I. Perlin D. S. (2017). Rapid and accurate molecular identification of the emerging multidrug resistant pathogen *Candida auris*. J. Clin. Microbiol. 55, 2445–2452. 10.1128/JCM.00630-17 28539346 PMC5527423

[B14] Kordalewska M. Lee A. Park S. Berrio I. Chowdhary A. Zhao Y.. (2018). Understanding echinocandin resistance in the emerging pathogen *Candida auris*. Antimicrob. Agents Chemother. 62, e00238–e00218. 10.1128/AAC.00238-18 29632013 PMC5971591

[B15] Kordalewska M. Perlin D. S. (2019). Identification of drug resistant *Candida auris*. Front. Microbiol. 10, 1918. 10.3389/fmicb.2019.01918 31481947 PMC6710336

[B16] Lockhart S. R. Etienne K. A. Vallabhaneni S. Farooqi J. Chowdhary A. Govender N. P.. (2017). Simultaneous emergence of multidrug-resistant *Candida auris* on 3 continents confirmed by whole-genome sequencing and epidemiological analyses. Clin. Infect. Dis. 64, 134–140. 10.1093/cid/ciw691 27988485 PMC5215215

[B17] Novak A. R. Bradley M. E. Kiser T. H. Mueller S. W. (2020). Azole-resistant *Aspergillus* and echinocandin-resistant *Candida* – What are the treatment options? Curr. Fungal Infect. Rep. 14, 141–152. 10.1007/s12281-020-00379-2 32699568 PMC7375389

[B18] Park J. Y. Bradley N. Brooks S. Burney S. Wassner C. (2019). Management of patients with *Candida auris* fungemia at community hospital, Brooklyn, New York, US –2018. Emerg. Infect. Dis. 25, 601–602. 10.3201/eid2503.180927 PMC6390749 30789336

[B19] Roberto A. Xavier D. E. Vidal E. E. Vidal C. Neves R. P. Lima-Neto R. G. (2020). Rapid detection of echinocandins resistance by MALDI-TOF MS in *Candida parapsilosis* complex. Microorganisms 8, 109. 10.3390/microorganisms8010109 PMC7023175 31940988

[B20] Sharma D. Paul R. A. Chakrabarti A. Bhattacharya S. Soman R. Shankarnarayan S. A.. (2020). Caspofungin resistance in *Candida auris* due to mutations in *Fks*1 with adjunctive role of chitin and key cell wall stress response pathway genes. bioRxiv. 10.1101/2020.07.09.196600

[B21] Valentin E. Tormo-Mas M. A. Eraso E. Pemán J. de Groot P. (2018). Molecular identification of *Candida auris* by PCR amplification of species-specific GPI protein-encoding genes. Int. J. Med. Microbiol. 308, 812–818. 10.1016/j.ijmm.2018.06.014 30025998

[B22] Vatanshenassan M. Boekhout T. Lass-Flörl C. Lackner M. Schubert S. Kostrzewa M.. (2018). MBT ASTRA: proof-of-concept for a rapid MALDI-TOF MS based method to detect caspofungin resistance in *Candida albicans* and *Candida glabrata*. J. Clin. Microbiol. 56, e00420–e00418. 10.1128/JCM.00420-18 30021820 PMC6113492

[B23] Vatanshenassan M. Boekhout T. Meis J. F. Berman J. Chowdhary A. Ben-Ami R.. (2019). *Candida auris* identification and rapid antifungal susceptibility testing against echinocandins by MALDI-TOF MS. Front. Cell. Infect. Microbiol. 9, 20. 10.3389/fcimb.2019.00020 30834236 PMC6387932

[B24] Vella A. De Carolis E. Vaccaro L. Posteraro P. Perlin D. S. Kostrzewa M.. (2013). Rapid antifungal susceptibility testing by matrix-assisted laser desorption ionization-time of flight mass spectrometry analysis. J. Clin. Microbiol. 51, 2964–2969. 10.1128/JCM.00903-13 23824764 PMC3754633

[B25] Vella A. De Carolis E. Mello E. Perlin D. S. Sanglard D. Sanguinetti M.. (2017). Potential use of MALDI-ToF mass spectrometry for rapid detection of antifungal resistance in the human pathogen *Candida glabrata*. Sci. Rep. 7, 9099. 10.1038/s41598-017-09329-4 28831086 PMC5567316

[B26] Wilkendorf L. S. Bowles E. Buil J. B. van der Lee H. A. L. Posteraro B. Sanguinetti M.. (2020). Update on matrix-assisted laser desorption ionization-time of flight mass spectrometry identification of filamentous fungi. J. Clin. Microbiol. 58, e01263–e01220. 10.1128/JCM.01263-20 32938733 PMC7685878

